# Efficacy of contralaterally controlled functional electrical stimulation compared to cyclic neuromuscular electrical stimulation and task-oriented training for recovery of hand function after stroke: study protocol for a multi-site randomized controlled trial

**DOI:** 10.1186/s13063-022-06303-y

**Published:** 2022-05-12

**Authors:** Jayme S. Knutson, Amy S. Friedl, Kristine M. Hansen, Mary Y. Harley, A. M. Barrett, Preeti Raghavan, Ela B. Plow, Douglas D. Gunzler, John Chae

**Affiliations:** 1 The MetroHealth System, Cleveland, OH USA; 2 Case Western Reserve University, Cleveland, OH USA; 3 Veterans Affairs Northeast Ohio Healthcare System, Cleveland, OH USA; 4 Emory University, Atlanta, GA USA; 5 Atlanta VA Healthcare System, Atlanta, GA USA; 6 Johns Hopkins University, Baltimore, MD USA; 7 Cleveland Clinic Foundation, Cleveland, OH USA

**Keywords:** Stroke rehabilitation, Hemiparesis, Upper extremity, Electrical stimulation, Therapy

## Abstract

**Background:**

Multi-site studies in stroke rehabilitation are important for determining whether a technology and/or treatment can be successfully administered by sites other than the originating site and with similar positive outcomes. This study is the first multi-site clinical trial of a novel intervention for post-stroke upper limb rehabilitation called contralaterally controlled functional electrical stimulation (CCFES). Previous pilot and single-site studies showed positive effects of CCFES on upper limb impairment and hand dexterity in stroke survivors. The main purpose of this study is to confirm and demonstrate the efficacy of CCFES in a larger group of most likely responders across multiple clinical sites.

**Methods:**

Up to 129 stroke survivors with moderate to severe upper extremity hemiparesis at 4 clinical trial sites will be randomized to CCFES, cyclic neuromuscular electrical stimulation (cNMES), or task-oriented-training (TOT). Participants will receive 12 weeks of group-specific therapy. Blinded assessments of upper limb impairment and activity limitation, quality of life, and neurophysiology will be used to compare outcomes at baseline, after treatment, and up to 6 months post-treatment. The primary endpoint is change in dexterity from baseline to 6 months post-treatment.

**Discussion:**

Loss of hand function following stroke is a major rehabilitation problem affecting millions of people per year globally. More effective rehabilitation therapies are needed to restore hand function in these individuals. This study will determine whether CCFES therapy produces greater improvements in upper extremity function than cNMES or TOT, and will begin to elucidate the different mechanisms underlying each of the three treatments. This multi-site study is a critical step in advancing a novel method of rehabilitation toward clinical translation and widespread dissemination.

**Trial registration:**

ClinicalTrials.gov NCT03574623. Registered prior to first enrollment; July 2, 2018.

**Supplementary Information:**

The online version contains supplementary material available at 10.1186/s13063-022-06303-y.

## Administrative information

Note: the numbers in curly brackets in this protocol refer to SPIRIT checklist item numbers. The order of the items has been modified to group similar items (see http://www.equator-network.org/reporting-guidelines/spirit-2013-statement-defining-standard-protocol-items-for-clinical-trials/).

**Table:** 

Title {1}	Efficacy of contralaterally controlled functional electrical stimulation compared to cyclic neuromuscular electrical stimulation and task oriented training for recovery of hand function after stroke: study protocol for a multi-site randomized controlled trial
Trial registration {2a and 2b}	NCT03574623 [ClinicalTrials.gov] [registered prior to first enrollment; July 2, 2018] https://clinicaltrials.gov/ct2/show/NCT03574623
Protocol version {3}	Version 3, 12-28-2020
Funding {4}	This research study is funded by the National Institutes of Health (NIH) National Center for Medical Rehabilitation Research (NCMRR) of the National Institute of Child Health and Human Development (NICHD), grant number R01HD092351. The stimulators used in this study were paid for, in part, from a Department of Veterans Affairs grant to the Cleveland Functional Electrical Stimulation Center. The use of REDCap is supported by a grant from NIH National Center for Advancing Translational Sciences (NCATS) to Case Western Reserve University, Clinical and Translational Science grant number UL1TR002548. The funders do not have a role in collection, analyzing, or interpreting data or in writing any manuscripts. The stimulators used in this study were manufactured by PDI Works LLC (Cleveland, Tennessee, USA) and are limited by federal law to investigational use.
Author details {5a}	• Jayme S. Knutson, jknutson@metrohealth.org, The MetroHealth System, Case Western Reserve University, and Veterans Affairs Northeast Ohio Healthcare System, Cleveland, Ohio, USA. • Amy S. Friedl, afriedl@metrohealth.org, The MetroHealth System, Cleveland, Ohio, USA. • Kristine M. Hansen, khansen1@metrohealth.org, The MetroHealth System, Cleveland, Ohio, USA. • Mary Y. Harley, mharley@metrohealth.org, The MetroHealth System, Cleveland, Ohio, USA. • A.M. Barrett, a.m.barrett@emory.edu, Emory University and Atlanta VA Health Care System, Atlanta, Georgia, USA. • Preeti Raghavan, praghav3@jhmi.edu, Johns Hopkins University, Baltimore, Maryland, USA. • Ela B. Plow, plowe2@ccf.org, Cleveland Clinic Foundation, Cleveland, Ohio, USA. • Douglas D. Gunzler, dgunzler@metrohealth.org, The MetroHealth System and Case Western Reserve University, Cleveland, Ohio, USA. • John Chae, jchae@metrohealth.org, The MetroHealth System and Case Western Reserve University, Cleveland, Ohio, USA.
Name and contact information for the trial sponsor {5b}	Investigator initiated clinical trial; Jayme S. Knutson (Principal Investigator) jknutson@metrohealth.org
Role of sponsor {5c}	This is an investigator initiated clinical trial. Therefore, the funders played no role in the design of the study and collection, analysis, and interpretation of data and in writing the manuscript.

## Background

Each year, approximately 795,000 Americans have a stroke [1]. After a stroke, loss of the ability to open one hand is common and is one of stroke’s most frequently persisting consequences [2]. Paresis of finger and thumb extensors profoundly affects upper limb function [3, 4], leading to significant disability and forcing many individuals to limit their preferences and participation to activities that do not require their affected hand, which diminishes quality of life [5]. Occupational therapy and relatively recent advances in rehabilitation (e.g., constraint-induced or robot-assisted therapies) are beneficial, but they are often limited in their effectiveness and applicability and are frequently difficult to deliver in a practical and cost-efficient way. Therefore, there remains a need for new therapies that can improve motor recovery across a wider range of severity of upper extremity impairment and can be practically administered. Because of this need, we designed and initiated the first multi-site study of a new therapy for post-stroke rehabilitation of the upper limb called contralaterally controlled functional electrical stimulation (CCFES).

CCFES therapy is based on neurophysiological principles of motor recovery after stroke. More than two decades of research has shown that post-stroke sensory and motor activities play an important role in motor recovery because the brain has inherent plasticity [6], that is, neuronal cortical connections and cortical representation areas, which were once thought to be static, are in fact modifiable by sensory input, experience, learning, and brain injury [7–14]. Activity-dependent modification of synaptic connections and reorganization of adult cortical areas may be explained by long-term potentiation (LTP) of excitatory postsynaptic potentials [7, 15]. LTP provides a molecular explanation of Hebb’s postulate that synapses are strengthened when pre- and postsynaptic neurons are repeatedly and synchronously active [16]. At higher levels of neural organization, Hebbian plasticity relates to the presence of temporally correlated neural activity [15]. Rehabilitation therapies that require or provide synchronous repetitive activation of neurons along motor and sensory pathways might facilitate synaptic remodeling, neural reorganization, and improved motor recovery. Therefore, therapies based on neurophysiological principles of motor recovery engage stroke survivors in active, repetitive, skill-requiring, goal-oriented movements.

**Fig. 1:**
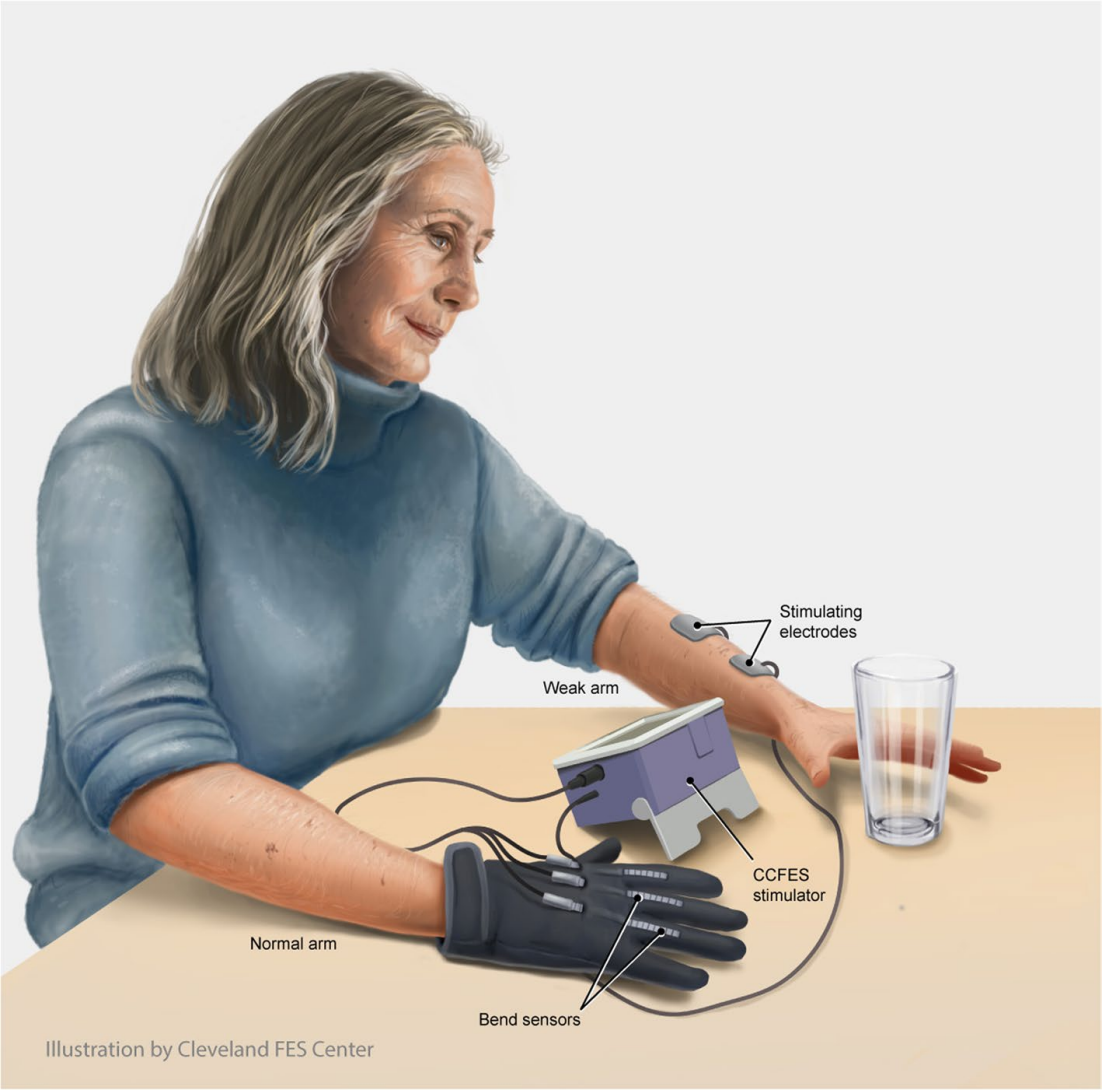
Contralaterally controlled functional electrical stimulation (CCFES) enables stroke survivors to open their paretic hand. The stroke survivor controls the amount of hand opening through a CCFES glove worn on their unaffected hand, which controls the intensity of electrical stimulation delivered to the finger and thumb extensor muscles of the paretic hand. The CCFES system enables stroke survivors to practice using their paretic hand to perform tasks in therapy and to self-administer hand opening exercises. Illustration by Erika Woodrum, CMI. © 2021 Cleveland FES Center, Cleveland, OH

CCFES is an innovative method of applying neuromuscular electrical stimulation (NMES). NMES of the paretic finger and thumb extensors is a common, practical, and inexpensive rehabilitation modality that can facilitate repetitive movement. But conventional cyclic NMES (cNMES) may have limited effectiveness because the stimulation is not controlled by the patient; therefore, the stimulated hand movement and corresponding proprioceptive feedback to the brain are not linked to (i.e., coincident with) the patient’s motor intention. CCFES is unique because the intensity of stimulation to the paretic extensors, and consequent degree of hand opening, are controlled by the patient through a glove instrumented with bend sensors worn on the unaffected hand (Fig. 1). With CCFES, volitional opening of the unaffected hand produces a simultaneous proportional degree of stimulated opening of the paretic hand. Patients are instructed to open both hands at the same time so that stimulation to the weak hand coincides with their active attempt to open it, which can give patients the perception that they have regained control of their hand. Thus, CCFES synchronizes motor intention (When I try to open my hand …) with the desired motor response (…my hand opens…) and the appropriate accompanying sensory experience (…and I see and feel it open). Furthermore, CCFES enables patients to use their paretic hand in therapy sessions (i.e., skill-requiring goal-oriented movement) even if they do not retain volitional hand opening, and to self-administer a high dose of CCFES-mediated hand opening exercise at home.

Since our first publication on CCFES in 2007 [17], we have conducted several studies demonstrating its feasibility and efficacy [18–21], and these positive outcomes of CCFES have been corroborated by other researchers [22, 23]. These studies provided strong preliminary evidence that CCFES therapy reduces upper limb impairment and improves hand dexterity more than an equal dose of cNMES, especially in patients who are less than 2 years post-stroke [18, 23]. A multi-site clinical trial is the next step in advancing this novel method of rehabilitation toward clinical acceptance, translation, and dissemination. The main objective of the multi-site study described in this article is to determine whether CCFES can be administered by therapists at sites other than the originating site with results confirming those of the prior single-site studies.

This study is designed to accomplish three specific aims. Aim 1 is to compare the effects of CCFES and cNMES in improving hand function after stroke. With cNMES, stimulation is delivered according to an on-off cycle, with the cycle timing, repetitions, and intensity of stimulation set by the therapist [24], thereby requiring no active participation from the patient. cNMES has been shown to reduce upper limb motor impairment compared to control groups [25, 26], although the longevity of effect and translation to functional improvements are inconsistent across studies [27]. Cyclic NMES is an ideal comparator to CCFES because it is widely used and therefore clinically relevant, but also because it will allow the study to determine the specific effects of CCFES and control for the effects of repetitive stimulated muscle contraction (e.g., conditioning/strengthening) as well as other non-specific factors that are not unique to CCFES. Thus, Aim 1 is an *explanatory* aim that will determine whether the method of delivering NMES actually matters. Aim 2 is to compare the effects of CCFES to task-oriented training (TOT), a high-quality structured post-stroke functional task practice therapy without electrical stimulation that includes a patient-administered home component dose-matched to the other two study treatments [28]. This is the first study that will compare CCFES to a therapy that does not include electrical stimulation but is similar in content to typical post-stroke occupational therapy. Therefore, Aim 2 is a *pragmatic* aim with great practical importance that increases the external validity of the study and will generate preliminary data for a future effectiveness trial. Aim 3 is an *exploratory* aim intended to generate preliminary data on the effects of CCFES, cNMES, and TOT on various measures of neurophysiology, in order to help elucidate and differentiate mechanisms of recovery associated with the treatments.

## Methods/design

### Overview

This is a parallel group, three-arm, single-blind, randomized controlled trial (RCT) of stroke survivors with upper extremity hemiparesis who are between 6 and 24 months past their stroke. A target of 129 participants will be randomized in equal proportions to CCFES, cNMES, or TOT. All groups receive 12 weeks of treatment consisting of a self-administered home exercise regimen and therapist-guided functional task practice in the research lab. Participants are followed for 6 months post-treatment. Blinded assessments take place at baseline (0 weeks), mid-treatment (6 weeks), end-of-treatment (12 weeks), and at 3 and 6 months post-treatment (24 and 36 weeks). The primary endpoint is change in dexterity at 6 months post-treatment, as assessed by the Box and Blocks Test (BBT).

There are 4 clinical sites participating in this study: MetroHealth Center for Rehabilitation Research (MCRR) and the Cleveland Clinic (Cleveland, Ohio, considered 1 site), Kessler Foundation (West Orange, New Jersey), Johns Hopkins University (Baltimore, Maryland), and Emory University and the Atlanta VA (Atlanta, Georgia, considered 1 site). MCRR is the lead site. The Cleveland Clinic is the only site conducting *exploratory* Aim 3 procedures and is not participating in Aim 1 or Aim 2 activities. Each site is conducting all procedures with human subjects according to ethical guidelines in conformance with the Declaration of Helsinki. Each site obtained approval to conduct the study from their site’s own Institutional Review Board. The study was registered on ClinicalTrials.gov under the title “Therapies for Recovery of Hand Function after Stroke,” National Clinical Trial number: NCT03574623.

### Participant recruitment and enrollment

Each site is responsible for recruiting and enrolling participants who meet the eligibility criteria established for the study. The trial sites employ strategies for recruitment that are best suited to their local environment and established referral patterns. A recruitment flyer template was created, and each site was encouraged to make it site-specific and obtain Institutional Review Board (IRB) approval for its use. Each site is encouraged to develop additional IRB-approved public or media-driven recruitment strategies. Potential study participants can find the study on ClinicalTrials.gov, where the contact information for each site’s study coordinator is listed. Recruitment from inpatient stroke and rehabilitation services, outpatient rehabilitation facilities, and outpatient clinics is encouraged. Each site has IRB approval of a HIPAA waiver that allows for subject pre-screening. Study coordinators at each site are encouraged to pre-screen medical records during acute stroke admission and inpatient rehabilitation at their clinical site. Potential participants identified early post-stroke may be contacted and, if agreeable, followed until they either do not meet the eligibility criteria or agree to participate in a formal eligibility assessment. Further pre-screening using an abbreviated list of the eligibility criteria is conducted by phone prior to a formal in-person eligibility assessment in which all eligibility criteria are evaluated. All study coordinators document and track their pre-screening activities using a phone screen log.

To encourage meeting recruitment goals, recruitment and enrollment progress is reviewed at monthly meetings attended by the principal investigator, site investigators, and study coordinators. In preparation for this monthly meeting, the site study coordinator at the lead site contacts the site study coordinators for an update on their number of pre-screens, in-person eligibility assessments, and enrollments. This site-specific data is presented in both tabular and graphical formats at the monthly meeting along with each site’s to-date enrollment goal. Challenges to enrollment and successful recruitment strategies are discussed at the monthly meetings, and emphasis is placed on encouraging and celebrating successful participant enrollment and promoting positive team spirit.

Potential study participants who pass the pre-screening and are interested in participating are mailed a consent form prior to their first in-person visit so they can review it themselves and with family members if desired. If they remain interested, a visit is scheduled to review the consent form, obtain informed consent, and assess eligibility. The study coordinator reviews the consent form with the candidate and with a caregiver present, if desired. If the coordinator questions the candidate’s capacity to consent, the site PI and/or study physician will be consulted. Discussion is encouraged during review of the consent form, and no eligibility assessment procedures are conducted before the consent form is signed. The study coordinators are instructed to be careful to avoid biasing the candidate towards or away from any of the three possible therapies they may receive as part of the study. The consent form describes the therapies using objective language and states that it is unknown which of the three treatments is better. The title of the study on the consent form is “Therapies for Recovery of Hand Function after Stroke” to help avoid biasing the participants toward or against any of the three therapies they may receive.

Once informed consent is obtained, the candidate is assessed for eligibility. Using a checklist of the inclusion and exclusion criteria (Table 1), the study coordinator evaluates each item. The site PI and/or physician are consulted if any of the eligibility criteria is in question. A manual of procedures elaborates on each eligibility criterion and provides specific instructions for evaluating them. Each site study coordinator has received training from the lead study coordinator to properly evaluate the eligibility criteria. Candidates whose eligibility is confirmed are given another opportunity to ask questions and decide whether to enroll. If they wish to proceed, the next two visits are scheduled for baseline assessments and treatment allocation/training, respectively. Also, candidates at the lead clinical site who pass pre-screening for participation in the Aim 3 procedures are scheduled for baseline neurophysiology assessments at the Cleveland Clinic, where a separate consenting process is undertaken pertaining specifically to the transcranial magnetic stimulation (TMS) procedures. Participation in the Aim 3 procedures is optional for individuals who are otherwise eligible for the study. A candidate is counted as enrolled and is assigned a participant ID if they return for baseline assessments and are subsequently randomized to one of the three treatment groups.

**Table 1:** Eligibility criteria

**Inclusion** 1. Age 21 to 90 years old at time of randomization 2. 6 to 24 months since a first clinical cortical or subcortical, ischemic, or hemorrhagic stroke 3. Unilateral upper limb hemiparesis with finger extensor strength of ≤4 out of 5 on the Medical Research Council scale 4. Score of ≥1 and ≤11 out of 14 on the hand section of the Upper Extremity Fugl-Meyer assessment (these define a degree of reduced hand strength and coordination that significantly limits use of the paretic hand in activities of daily living) 5. Able to follow 3-stage commands 6. Able to recall at least 2 of a list of 3 items after 30 min 7. Adequate active movement of shoulder and elbow to position the paretic hand in the workspace for table-top task practice 8. Skin intact on hemiparetic arm 9. Surface electrical stimulation of the paretic finger and thumb extensors produces functional hand opening without pain (this will exclude individuals who have too much flexor spasticity) 10. Able to hear and respond to cues from the stimulator 11. Not receiving occupational therapy 12. Full volitional hand opening/closing of contralateral hand 13. Demonstrates ability to follow instructions for putting on and operating the assigned stimulator or has caregiver to assist (if applicable) **Exclusion** 1. Concomitant neurologic diagnosis of peripheral nerve injury, Parkinson’s disease, spinal cord injury, traumatic brain injury, or multiple sclerosis 2. Brainstem stroke 3. Uncontrolled seizure disorder, defined as having a seizure within the previous 3 months	4. Severe shoulder or hand pain, i.e., unable to position hand in the workspace without pain 5. Lack of functional passive range of motion of the wrist or fingers of affected side 6. Insensate to touch on forearm or hand 7. Uncompensated hemi-neglect (extinguishing to double simultaneous stimulation) 8. History of potentially fatal cardiac arrhythmias with hemodynamic instability 9. Cardiac pacemaker or other implanted electronic system 10. Diagnosis (apart from stroke) that substantially affects paretic arm and hand function 11. Deficits in communication that interfere with reasonable study participation 12. Lacking sufficient visual acuity to see the stimulator’s display 13. Botulinum toxin injections to any upper extremity muscle within 3 months of enrolling 14. Pregnancy 15. Concurrent enrollment in another investigational study **Additional exclusion criteria for participating in the neurophysiological assessments (Cleveland site only)** 16. Metal in the head or neck 17. Conditions that affect sensorimotor system: brain tumor(s), diabetic neuropathy, Parkinson’s disease 18. Seizure history or history of fainting spells of unknown/undetermined etiology 19. Alcohol or substance abuse disorder 20. Deep brain stimulator, shunts, nerve stimulators, pace-making, or pace-recording devices 21. Current diagnosis of carpal tunnel syndrome 22. Previous adverse reaction to transcranial magnetic stimulation (TMS) 23. Medications with potential to alter seizure threshold, e.g., buproprion

Throughout the recruitment and enrollment process, participant characteristics are collected so that the balance of covariates across groups and their effects on the outcomes can be evaluated at the end of the study. Each participant is characterized with respect to demographic (e.g., age, sex, race, ethnicity, pre-morbid hand dominance), stroke-related (e.g., time post-stroke, hemorrhagic or ischemic, cortical or subcortical, right or left hemisphere), and neurological variables (e.g., side of hemiparesis, cognition, aphasia), as well as co-morbidities, and medications taken.

### Assessments

Assessments are conducted by trained occupational or physical therapists at each site who are blinded to which treatment group the participant is in. The assessments take place at 5 time points: baseline (0 weeks), mid-treatment (6 weeks), end-of-treatment (12 weeks), and at 3 and 6 months post-treatment (24 and 36 weeks, respectively) (Table 2). To reduce the occurrences of missing data, the assessing therapist calls the participant 1 to 2 days before the assessment visit to remind them of the time and place of the upcoming visit. If the participant is still within the 12-week treatment phase of the study, the treating therapist reminds the participant to refrain from any electrical stimulation (cNMES or CCFES groups) or study therapy (TOT group) for 24 h prior to their mid-treatment and end-of-treatment assessment visits. This is to prevent the outcomes from being affected by any transitory effects of the treatments. The assessments are conducted in a quiet setting with participants seated in a comfortable chair with no armrests. If needed, rest breaks are permitted throughout the assessment visit.

**Table 2:** Schedule of enrollment, interventions, and assessments

Study period	Pre-treatment	Treatment			Follow-up	
**Timepoint (weeks)**	**<− 2**	**0**	**6**	**12**	**24**	**36**
**Enrollment**						
Informed consent	X					
Eligibility assessment	X					
Baseline variables		X				
Allocation		X				
**Interventions**						
CCFES		**◊------**	**-------------**	**------◊**		
cNMES		**◊------**	**-------------**	**------◊**		
TOT		**◊------**	**-------------**	**------◊**		
**Assessments**						
End of Treatment Questionnaire				X		
SIS – Hand Section		X		X		X
Neuro-QOL UE Function		X		X		X
Modified Ashworth Scale		X	X	X	X	X
UEFM		X	X	X	X	X
BBT		X	X	X	X	X
SULCS		X	X	X	X	X
ARAT		X	X	X	X	X
MEP excitability		X		X		X
Ipsilateral MEP excitability		X		X		X
Interhemispheric inhibition		X		X		X

*CCFES* contralaterally controlled functional electrical stimulation, *cNMES* cyclic neuromuscular electrical stimulation, *TOT* task-oriented-training, *SIS* stroke impact scale, *QOL* quality of life, *UE* upper extremity, *UEFM* upper extremity Fugl-Meyer assessment, *BBT* box and blocks test, *SULCS* stroke upper limb capacity scale, *ARAT* action research arm test, *MEP* motor evoked potential

The primary endpoint is change in dexterity from baseline to 6 months post-treatment, as assessed by the Box and Blocks Test (BBT). This is the outcome measure and time point that showed the largest effect in our previous single-site RCT [18]. The secondary endpoint is the response rate on the BBT at 6 months post-treatment, defined as the proportion of participants with a change of 8 points or more. Secondary measures of upper extremity motor recovery include the Upper Extremity Fugl-Meyer (UEFM) assessment, Stroke Upper Limb Capacity Scale (SULCS), and Action Research Arm Test (ARAT). These measures are described below. A full list of assessments, and the order in which they are conducted, is shown in Table 2. Neurophysiology assessments are conducted on a separate day from the others. The full battery of assessments is intended to evaluate multiple domains of the International Classification of Functioning and Disability Framework (ICF) [29], including activity limitation, impairment, and quality of life.

#### Box and Blocks Test (BBT)

The BBT is a measure of manual dexterity, which requires the subject to pick up one block at a time, move it over a partition, and release it in a target area as many times as possible in 60 s. The BBT is classified as an activity (ICF domain) measure by the Rehabilitation Measures Database [30] and the Evidence-Based Review of Stroke Rehabilitation [31]. Since the BBT measures functional grasp and release, it is an appropriate primary outcome measure for this study since the treatments focus on restoring functional hand opening and closing. The BBT is also relatively easy to standardize in its administration and scoring, compared to alternative motor impairment or function measures. Norms have been established for various populations including healthy elderly individuals [32, 33]. The minimum detectable change (MDC) has been determined as 5.5 blocks [34]. In the absence of an established minimal clinically important difference (MCID), 5.5 (round up to 6) may be a reasonable choice for MCID and is being used by others [35], but to be conservative a gain of 8 blocks was chosen as a clinically important difference in this study, which is a healthy margin above the MDC and slightly greater than 10% of the age-adjusted norms. Participants with moderate impairment in our previous study who had an 8 point gain or greater on the BBT, also had an average gain of 4.7 on the UEFM, which is greater than the 4.25 MCID for UEFM [36] and supports the use of 8 points on the BBT as a cutoff for clinical significance.

#### Upper Extremity Fugl-Meyer Assessment (UEFM)

The UEFM [37] is a reliable and valid measure of post-stroke upper limb motor impairment [38]. The UEFM items take into account synergy patterns, isolated strength, coordination, and hypertonia. Volitional movement of the upper limb (shoulder, elbow, forearm, wrist, and hand) is examined in and out of synergies. Each item is graded on a 3-point ordinal scale (0, cannot perform; 1, perform partially; and 2, perform fully) and summed to provide a maximum score of 66.

#### Stroke Upper Limb Capacity Scale (SULCS)

The SULCS is a measure of capacity, the ability to execute a task in a standardized setting. It was designed to be easier to score and less time consuming than other existing capacity measures [39]. The SULCS consists of 10 tasks that relate to daily activities. Each receives a score of 0 (unable to perform) or 1 (able to perform), resulting in a total score of 0–10. The SULCS is a valid and reliable measure that is suitable for a wide range of patients including those with poor hand-related upper limb capacity [40]. The SULCS has been shown to strongly correlate with the Arm Motor Ability Test (AMAT) (*ρ* = 0.81–0.93), a measure of capacity used in our prior CCFES RCT [18]. Because the SULCS can be administered much quicker than the AMAT (6 min vs. >20 min) [41], it was chosen for use in this study.

#### Action Research Arm Test (ARAT)

The ARAT is a 19-item observational measure used to assess upper extremity motor function in stroke recovery [42]. Items comprising the ARAT are categorized into four subscales (grasp, grip, pinch, and gross movement) and arranged in order of decreasing difficulty, with the most difficult task examined first, followed by the least difficult task. Task performance is rated on a 4-point scale, ranging from 0 (no movement) to 3 (movement performed normally), with a maximum score of 57. The ARAT has high/excellent test-retest reliability and interrater reliability [43, 44]. It is widely used in upper extremity stroke rehabilitation research and will therefore provide some basis for comparison of the findings of this study to those of other studies.

#### Other assessments

Additional secondary measures include change in muscle tone of the wrist and finger flexors using the Modified Ashworth Scale [45], quality of life (QOL) using the Hand Section of the Stroke Impact Scale (SIS) [46] and the Neuro-QOL Upper Extremity Function (Fine Motor, ADL) [47] questionnaires, and impressions of the effectiveness, dose, and ease of doing the home regimen via an End-of-Treatment Questionnaire administered by the study coordinator, who is not blinded.

The neurophysiology assessments associated with exploratory Aim 3 include contralateral motor evoked potential (cMEP) excitability, ipsilateral MEP (iMEP) excitability, and interhemispheric inhibition (IHI). All of these assessments are made by applying single-pulse TMS and recording surface electromyographic (EMG) signals from finger extensor muscles (extensor digitorum communis, EDC) as a means of quantifying changes in corticospinal and interhemispheric connectivity. cMEP and iMEP measure physiologic excitability of crossed and uncrossed corticomotor pathways the paretic EDC, respectively. IHI measures the inhibitory effect of the contralesional primary motor cortex upon ipsilesional primary motor cortex conducted via transcallosal connections. An ipsilateral silent period method is used to measure IHI, where TMS is delivered to contralesional primary motor cortex during tonic voluntary contraction of paretic EDC. Transient suppression of EDC EMG is taken as an index of IHI imposed from the contralesional motor cortex. The procedures being used for these measurements are well established [48–50].

#### Changes in concomitant therapy or medication

This study only enrolls participants who are no longer receiving rehabilitation therapies to the upper limbs. Once enrolled, the use of electrical stimulation or other experimental rehabilitation techniques involving the hemiparetic upper limb, changes in spasticity medications, botulinum toxin injections to upper limb muscles, shoulder steroid injections, use of an arm sling, and use of a resting hand splint is discouraged unless it is deemed medically necessary. Physical therapy that does not target the upper extremity and speech therapy are not discouraged. To account for the potential confounding effects of such concomitant therapies, at each assessment visit, the assessing therapist asks the participant if they have received any other therapies or interventions focused on their paretic upper limb since their last visit and if there have been any changes in medication prescriptions or dosages. The classes of medications documented and tracked in this study include the following: alpha agonists, alpha-1 antagonists, antidepressants, anti-seizures, anti-spasticity, sedatives/hypnotics/anxiolytics, and neuroleptics/antipsychotics. Participants who choose to receive concomitant therapies while they are in the treatment phase will be asked to complete the treatment and follow-up periods. Their data will be excluded from the per-protocol analysis but will be included in the intent-to-treat analysis.

#### Standardization of assessments

All sites are supplied with manuals, scoring criteria, and the standardized kit of equipment for administering the BBT, UEFM, SULCS, and ARAT. All assessing therapists undergo training and an initial certification process prior to conducting any assessments. The training is provided by the lead site’s assessing therapist via teleconferencing and includes instruction and demonstration of how to administer the BBT, SULCS, Modified Ashworth Scale, and questionnaires (SIS and Neuro-QOL). Training on the UEFM and ARAT is provided online using materials developed in the lab of Steven C. Cramer (University of California, Los Angeles), and administered online by Blue Cloud (www.healthcarepoint.com). In addition to initial training, each assessing therapist must be certified to administer BBT, UEFM, SULCS, and ARAT and re-certified yearly on the ARAT and UEFM. Initial certification on BBT and SULCS requires the assessing therapist to view two videos of stroke survivors performing the BBT and the SULCS, and to score the BBT and SULCS correctly as determined by the assessing therapist at the lead site. Initial certification and re-certification on the UEFM and ARAT is done through the Blue Cloud online training which entails presentation of case studies, videos of how to score the assessments, and tests. Initial certification and re-certification requires the assessing therapist to score greater than 95% on the tests. All current certificates and all documentation of training, certification, and re-certification are stored, and expiration dates are tracked by the study coordinator at each site and reported to the lead site. Reminders of times to re-certify are given at monthly team meetings.

### Randomization

After baseline assessments, subjects are assigned to a treatment group using an adaptive randomization algorithm [51, 52] to minimize group imbalances on 4 key participant characteristics: (1) time post-stroke (<15 months vs. ≥15 months), (2) age at randomization (<58 years vs. ≥58 years), (3) moderate vs. severe impairment, and (4) study site. Fifteen months post-stroke was chosen as the stratification cutoff because it is the middle of the 6- to 24-month post-stroke eligibility time-window. Age 58 was the average age of participants in the previous study. “Moderate” is defined as having at least 10° active wrist, thumb, and finger extension in at least two digits [53]. Less movement than this is considered to be “severe” impairment. During baseline assessment, the assessing therapist at any clinical site enters the above four participant characteristics into an online REDCap data collection form created for this study. This action triggers REDCap to automatically send an email to the study coordinator at the lead site informing her that a new participant is ready to be randomized. She then accesses the computer program (Matlab, The Mathworks, Inc., Natick, MA) running the minimization algorithm, enters the stratification variables, and the program outputs the treatment group assignment and participant ID. She then enters the group assignment and participant ID into a REDCap form that can be accessed by the participant’s treating therapist.

### Interventions

For all participants, the intervention lasts 12 weeks and consists of the following: (1) 22 sessions of therapist-guided functional task practice in the clinical research lab (two per week except on weeks 6 and 12, which include an assessment visit), and (2) 10 sessions per week of a self-administered exercise regimen at home (Table 3).

**Table 3:** Treatment regimen for each group

Group	Lab Task Practice (22 sessions in 12 weeks)	Home Exercise (10 sessions/week)
CCFES	Observe home regimen (15 min). CCFES-mediated functional task practice (75 min)	CCFES-mediated cued hand opening (1 session = 22-min period × 2)
cNMES	Observe home regimen (15 min). Functional task practice with no stim (75 min)	cNMES-mediated hand opening (1 session = 29-min period × 2)
TOT	Review home regimen (15 min). Functional task practice – same as cNMES (75 min)	TOT home exercise program (1 session = 30-min period × 2)

#### Electrical stimulator set up and participant training

At the next visit after baseline assessments, the treating therapist informs the participant of their treatment assignment and instructs them on how to perform the home exercise component of the interventions. For participants in the CCFES and cNMES groups, this visit starts with a procedure for determining electrode placements and setting up the stimulator. All treating therapists are trained to perform these procedures by the treating therapist at the lead site and are provided a manual to guide them.

The stimulator used in this study is a custom-designed device with a 4.25-in. touchscreen interface developed by the lead site and manufactured by PDI Works, LLC, Cleveland, TN. It is an investigational device that has received FDA approval for use in this study as a Non-Significant Risk study. The stimulator delivers biphasic rectangular current pulses at a pulse frequency of 35 Hz. For this study, 40 mA is the default pulse amplitude setting, but 60 mA may be used if needed to achieve hand opening, and up to three channels of stimulation are used. Surface electrodes (2” × 2” square and 1.25” round pre-gelled self-adhesive) are positioned to activate the finger and thumb extensor muscles, primarily the extensor digitorum communis and extensor pollicis longus, but also the abductor pollicis brevis, dorsal interossei, or extensor indicis proprius if they are needed to achieve functional hand opening [20]. A maximum stimulus intensity (pulse duration) is determined for each stimulus channel so as to achieve maximum finger and thumb extension without discomfort. Using the touchscreen interface, the treating therapist selects cNMES or CCFES, according to the participant’s group assignment. When cNMES is selected, the stimulus channels are programmed so that stimulation intensities (pulse durations) increase and decrease according to the timing option the therapist selects within the exercise regimen menu. When CCFES is selected, the stimulus channels are programmed so that stimulation intensities change in proportion to the amount of opening of a CCFES glove worn on the unaffected hand. The CCFES glove is an assembly of 3 bend sensors attached to the fingers of a common work glove [17]. Finally, the therapist sets the duration of exercise periods according to group assignment and the cue timing (described below) that is issued during exercise sessions.

After completing the stimulator setup (cNMES and CCFES groups), the treating therapist instructs and trains the participants (and their caregivers if necessary) how to put on the components (i.e., electrodes, glove if applicable). Digital photographs of the electrodes correctly positioned on the arm are taken, printed out, and sent home with the participant to assist them in placing them correctly at home. Electrodes are replenished on a weekly basis regardless of wear. The therapist and participant review a folder containing a group-specific manual that gives step-by-step instructions on how to operate the stimulator, complete a home exercise session, and document each home exercise session in a diary. Before being sent home with the stimulator, the participants demonstrate that they can put on the electrodes and glove (if applicable) and start an exercise session. Participants in the TOT group (no stimulator) receive a kit of equipment for hand exercises and tasks (e.g., tennis ball, cup, thera-putty, blocks, buttoned shirt) and are trained how to use a TOT-specific manual (described below) to guide them in practicing specific exercises for certain durations at home. The therapist has the participant complete one or two repetitions of each exercise they prescribe and ensures they understand how to use the TOT manual and how to document their home exercise sessions in a diary.

#### Therapist-directed lab sessions

At the lab sessions, for all groups the treating therapist observes the participant performing 15 min of the regimen they do at home (described below) to reinforce instructions and troubleshoot any problems. Then for 75 min, the therapist instructs and guides the participant in practicing functional tasks with their paretic hand. Tasks involve using the paretic hand to pick up, manipulate, and release objects commonly used in daily life. Within a session and over the course of multiple sessions, the therapist continually adjusts the difficulty of tasks, increasing the difficulty level from easy-to-acquire-and-manipulate tasks to tasks requiring wider hand opening, greater complexity and skill, graded hand opening (release), and coordination of hand function with proximal upper limb movement. The pace and content of the therapy is standardized via study-specific protocols and task lists (see below). For subjects with severe hand impairment, early task practice sessions focus on simpler tasks, such as opening the hand adequately to acquire a small object. If it is not possible to perform tasks, then the therapist focuses on passive and active-assisted range of motion, arm placement activities in preparation for hand tasks, and hand placement on target surfaces and objects.

The CCFES group uses CCFES to assist the paretic hand in performing tasks. The cNMES group practices tasks using their paretic hand but without any stimulation because cNMES is not controlled by the participant. Therefore, to ensure that the two stimulation groups receive an equal weekly duration of electrical stimulation (~10 h/week), the duration of the home stimulation exercise sessions is longer for the cNMES group than for the CCFES group (Table 3). The TOT group receives the same task practice as the cNMES group (i.e., with no electrical stimulation).

#### Participant-administered home sessions

Home sessions are self-administered by the participant. For all three groups, the participants are instructed to distribute the 10 prescribed self-administered home sessions per week so that no more than two sessions are done per day and they are separated by at least 2 h.

Each home session lasts 47 min for the CCFES group and 61 min for the cNMES group (including a 3-min rest break), so that the total duration of stimulation received by both groups is equivalent, ~10 h per week (CCFES: 44 min per home session × 10 home sessions per week + 90 min per lab session × 22 lab sessions in 12 weeks ≈10 h per week; cNMES: 58 min per home session × 10 home sessions per week + 15 min per lab session × 22 lab sessions in 12 weeks ≈10 h per week). During an exercise session, participants with CCFES are prompted by audio and visual cues issued from the stimulator to repeatedly open *both* hands for 6 s and then relax for 14 s. These cue durations are used for weeks 1 and 2, and changed by the treating therapist to 8 s open and 12 s relax for weeks 3 and 4, and then to 10 s open and 10 s relax for weeks 5 to 12. This graduated schedule is used to prevent muscle fatigue early in the 12-week intervention and to build greater strength with longer duration muscle contractions as the treatment progresses. For participants with cNMES, the stimulator automatically ramps on and off with the same timing as the cues that are produced for CCFES, and the participants are instructed to attempt to open their paretic hand when the stimulation ramps on and relax the paretic hand when the stimulation ramps off in synchrony with audio and visual cues. The cNMES group is also instructed to keep the non-paretic hand still during exercise sessions (i.e., do not open and close). Once a cNMES or CCFES participant starts an exercise session using the touchscreen interface, the CCFES cues or automatic cNMES stimulation cycles continue according to the timing set by the treating therapist until the session reaches its end or the participant ends the session early and turns the device off.

For the TOT group, a home exercise session consists of two 30-min periods separated by 3 min of rest. To standardize the home exercise regimen for the TOT group, the Graded Repetitive Arm Supplementary Program (GRASP) [28, 54] was adapted for this study. GRASP is a self-administered arm and hand exercise program that has been shown to improve arm and hand function, grip strength, and amount of use [28]. Adaptations of the GRASP manuals (participant and clinician) were created with permission, and TOT participants are instructed by their treating therapist on how to use the manual to guide their home exercise regimen. The TOT participant manual is a 46-page guide with photographs and easy-to-follow instructions on how to perform 35 specific arm and hand exercises. The exercises are grouped into 5 sections within the manual: stretching, arm strengthening, hand strengthening, coordination, and hand skills. Most of the exercises have options for progressing the number of repetitions and/or degrees of difficulty. The manual starts with simple exercises and progressively guides a participant through exercises and tasks that require greater coordination and motor skill. The manual emphasizes the importance of doing repetitions of movements and of using the stroke affected arm as much as possible in daily life. An exercise session is comprised of 1 h (plus a 3-min rest halfway through) working through a portion of the manual or the entire manual as prescribed by the treating therapist, who also prescribes an appropriate degree of difficulty and/or repetitions for each exercise. The clinician’s manual gives guidance for how and when to increase the difficulty of the exercises.

#### Adherence monitoring

Adherence to the home stimulation regimen is monitored at every lab session during the treatment period. For participants in the CCFES and cNMES groups, the stimulator logs the date and time the unit is turned on and off and the number of exercise sessions completed. In addition, participants in all three groups are given a diary sheet each week to log completion of home sessions. At each lab visit, the treating therapist checks the diary sheet and discusses the number of sessions logged since the last treatment session, and in the case of CCFES and cNMES participants, compares it to the electronic data logs from the stimulator. The treating therapist continually encourages compliance with the home exercise program at each visit. Also, attendance at scheduled lab sessions is documented so that adherence to lab sessions can be determined.

#### Standardization of interventions

Several methods are used to standardize the pace and content of the lab functional task practice sessions across treating therapists and sites.

1. Each treating therapist receives initial training from the treating therapist at the lead site via teleconferencing. This training includes approximately 3 h of instruction and discussion of all 3 treatments, demonstrations of how to position electrodes and set up the stimulator for the CCFES and cNMES groups, and review and discussion of study documents that define the interventions and serve as guidance to the treating therapist.
2. A manual of procedures (MOP) was created, which gives instructions for how to structure and segment functional task practice sessions, provides principles for what should constitute a task and what compensatory strategies should be allowed or discouraged, describes how to instruct CCFES participants how to use CCFES during task practice, and gives guidance on how much instruction and cueing to provide participants as they attempt and repeat tasks.
3. A standardized kit of items to use for the lab task practice sessions was sent to each site, and additional guidance documents were created to further standardize the content of functional task practice sessions. These include lists of functional tasks arranged under activity categories (e.g., eating, office, kitchen, laundry) along with guidance for choosing appropriate activity categories and tasks according to participants’ interests and the severity of their motor impairment, while encouraging treating therapists to use their clinical judgement and experience to create and adapt specific tasks and to determine when to make an activity more challenging and how to instruct the participant to perform the tasks successfully.
4. To help standardize the dose and pace of functional task practice sessions, the treating therapists are instructed to provide 75 min of therapy divided into five 15-min periods, each period working on a different task. This allows time for problem solving to determine the best approach to accomplish the task and for multiple repetitions for motor learning. A specific number of repetitions per task is not prescribed since this will be dependent participant factors such as degree of impairment; however, to help standardize dose and pace, for participants with baseline UEFM score ≤28 a minimum of 15 repetitions per task is suggested, and for participant with baseline UEFM score >28 at least 30 repetitions per task is suggested.
5. The above content and pacing guidance has been embedded into the study’s REDCap database, which also serves as a real-time guide that the treating therapist uses during the treatment sessions. The REDCap treatment visit form prompts the treating therapist to choose tasks and document the content of every treatment session, including which tasks practiced, utensils/tools used during the task, and number of task repetitions.
6. Treating therapists are initially certified by satisfactorily completing the training provided by the lead site’s treating therapist. They must be re-certified every 3 months during their first year on the study, every 6 months during their second year, and once a year thereafter. Re-certification requires the treating therapist to submit a video of themselves administering functional task practice to a participant. The video is evaluated by the treating therapist at the lead site who provides feedback and completes a certification form. Reminders to re-certify are sent up to 4 weeks before they are due.

### Blinding considerations

In this study, the assessing therapists are blinded to treatment assignment; it is not possible to blind the treating therapists or participants. To help maintain blinding, the treatment visits are held at a location where assessing therapists will not be aware of the treatment being provided or behind closed doors. Also, at each site, the assessing therapist performs their testing in a different room than where the treating therapist interacts with study participants, and, for each participant, assessments take place on different days than the treatment visits. Prior to the mid-treatment (6 weeks) and end-of-treatment (12 weeks) assessment visits, the treating therapist reminds the participant to not disclose their treatment group to the assessing therapist. Participants are instructed to not bring any study materials (e.g., the stimulator or manuals) to the assessment visits. At the beginning of each assessment visit, the assessing therapist again reminds the participant to not disclose anything about the study treatment they received or are receiving.

In the event that unblinding does occur, the assessing therapist documents that they were unblinded in the study database. To help assess the success of blinding, at the end of each assessment visit the assessing therapist fills in a questionnaire asking if they believe they are still blinded or not and which treatment they think the participant has received. Since study investigators are not blinded, there is no anticipated need or planned procedures for unblinding an assessing therapist.

### Retention

Participants may discontinue the study at any time for any reason if they wish to do so without any consequences, as stated in the consent form. The participant’s involvement may also be ended by the investigators if the participant does not follow instructions, misses scheduled visits and cannot be contacted, or if their medical condition changes and increases their risk of adverse events. Participant data that have been collected up to the time of their discontinuation will be included in the analysis.

To facilitate retention, study staff clearly communicate the time commitment and expectations at the outset of the study. The consent form very clearly describes with text and graphics the phases of the study and their durations, the number and duration of treatment and assessment visits, and the frequency and duration of the home exercise sessions. The treating therapist works with the participant to create a full schedule of visits during the first visit after the baseline assessment, and this schedule is placed in the participant’s folder that they take home and return with at each treatment visit. The participant’s diary also serves as a calendar that shows them when they have treating and assessing visits and when they have home exercise sessions to do. Contact information is exchanged so that study staff have multiple means of contacting the participant, and the participant has the contact information of multiple study staff. The frequency of visits during the treatment phase (twice a week) and professional relationship the treating therapist builds with the participant during those visits helps minimize missed treatment visits. Reminder calls are made prior to each assessment visit. In addition, the study provides transportation to the study visits at no cost to the participant or compensates the participant for mileage and parking if they drive themselves or have a caregiver give them a ride. For those requiring transportation, the study staff makes all the arrangements with a transportation company, making it as easy as possible for the participants to come to treatment visits. Finally, to facilitate adherence with the follow-up visits, an incentive payment of $50 is given to participants for completing the 3- and 6-month follow-up visits (up to $100 total).

### Data management

Study data are collected and managed using REDCap electronic data capture tools hosted at Case Western Reserve University [55, 56]. REDCap (Research Electronic Data Capture) is a secure web-based software platform for building and managing online databases and surveys. Every study visit has an accompanying electronic form in REDCap that is to be filled in by the appropriate study staff person (e.g., study coordinator, treating therapist, assessing therapist). The REDCap forms guide the research team members through each visit’s procedures, and thereby help ensure that procedures are done consistently across sites, that all outcome and treatment data are being collected, and that ancillary events that may impact the study (e.g., adverse events, protocol deviations, reasons for missing data) are being documented. Data are directly entered into the appropriate REDCap form at the study visit. If paper forms (i.e., printouts of the REDCap forms) need to be used, data are transcribed from them into the REDCap database. REDCap also serves as the centralized repository of study guidance documents (e.g., the manual of procedures), so they are easily accessible across sites. In addition to using REDCap for data storage, a hard-copy file folder is kept for each participant to store paper documents containing protected health information, such as signed consent forms, copies of medical records, and radiology reports. These are securely maintained by the study staff at each site according to their institutional guidelines.

### Statistical analysis

#### Sample size and power

The primary endpoint is change in BBT score from baseline to 6-month post-treatment. Based on data from a previous single-site study [18], the estimated CCFES vs. cNMES effect size in participants who are <2 years post-stroke is 0.64 (Cohen *d*). With this effect size, a type I error of .05, and 80% power, 39 participants per group are needed in the CCFES and cNMES groups. Although it is hypothesized that the treatment effect between CCFES and TOT is larger than between CCFES and cNMES, to be conservative, the same sample size of 39 is the goal for the TOT group for comparison with CCFES. With an expected attrition rate of 10%, 129 is the planned sample size to ensure 39 per group complete the study. The secondary endpoint is the response rate on the BBT at 6-month post-treatment, defined as the proportion of participants with a change of 8 pts or higher at 6-month post-treatment. Based on data from the previous study, the estimated response rate for the target population is 45% for CCFES and 8% for cNMES, a between-group difference of 37%. With an *N* of 129, this trial is powered sufficiently to detect a minimum of 29% difference in response rate between groups.

#### Data analysis plan

Initial analyses will be performed by the study statistician using the intent-to-treat principle and include all randomized participants, comparing outcomes by assigned group. A secondary per-protocol analysis will include all participants who completed at least 80% of their assigned treatment (i.e., at least 80% of their functional task practice visits and at least 80% of their assigned home exercise sessions). Data will be analyzed at the end of the study; there are no interim analyses planned.

Data will be analyzed for patterns of missing data, and appropriate methods of imputation will be used to account for missing data from loss to follow-up or missed outcome assessments. Changes from baseline in BBT, UEFM, SULCS, ARAT, and SIS scores will be modeled using a linear mixed effects approach which is well-suited for handling correlated repeated measurements, unbalanced data, and missing data and dropouts in longitudinal studies, while permitting control for potential confounders [57]. This approach allows estimates and comparisons of changes from baseline at any time point and also allows comparisons and estimates across time points within groups to evaluate the persistence or decay of the treatment effect. Exploratory looks at the data will be done before fitting our models. The adaptive randomization should force relatively good balance of potential confounding variables (e.g., time post-stroke, age, severity of impairment, investigational site), but imbalances in treatment adherence or any other baseline variable (e.g., handedness, lesion location) may still require their inclusion as covariates. Poolability of data across sites will be evaluated and adjusted for as needed. Treatment by site interaction will be tested.

For Aims 1 and 2, the entire sample, including both moderate and severe participants, will first be analyzed. Based on our previous study, the inclusion of participants with severe hand impairment is expected to dilute the treatment effects, especially on the BBT. Therefore, we will also analyze the participants with moderate hand impairment at baseline separately from those with severe impairment. We will also evaluate the effects of additional potential moderators (e.g., time post-stroke, adherence, lesion location (cortical vs. subcortical)) in separate models, first using regression analyses to determine if treatment response is associated with any of these potential moderators [58].

For the primary endpoint, BBT change from baseline to 6 months post-treatment, the adjusted least square means estimated from the linear mixed effects models will be compared between the CCFES and cNMES groups. Adjusted least square means and the associated 95% confidence interval will be presented as well as the *p*-value. The sample size is based on this endpoint with a type I error of 0.05, thus, a *p* ≤.05 will be used to declare significance. For the secondary endpoint, response rate on the BBT will be calculated for the CCFES and cNMES groups and compared using a logistic regression modeling approach. Similar analyses will be used to analyze the UEFM, SULCS, ARAT, SIS scores, and other secondary measures.

Aim 3 (comparing changes in neurophysiology measures among the 3 groups) will follow the analyses of Aims 1 and 2 to evaluate between-group differences in changes in the TMS-derived neurophysiological measures, e.g., reduction in interhemispheric inhibition and increase in iMEP excitability from contralesional motor cortex. In addition, linear regression models will be used to evaluate associations between neurophysiology outcomes and motor outcomes (SULCS, ARAT, BBT, UEFM). As an exploratory aim, conclusions from Aim 3 will be tentative, and we will look for reproducibility in future studies.

### Adverse event monitoring

All study staff are trained to monitor and document adverse events (AE), defined as any untoward or unfavorable medical occurrence in a participant, including any abnormal sign, symptom, or disease, occurring while the participant is enrolled in the study, regardless of whether the event is considered related or unrelated to the participant’s involvement in this research. The REDCap database for this study includes AE report forms that guide the research staff in reporting AEs. In particular, the report includes the date the AE occurred, when in the course of the study the AE occurred, a description of the AE using the SOAP format (Subjective, Objective, Assessment, Plan), and the reporting staff’s opinion as to whether the AE (a) is unexpected or not, (b) is related or possibly related to the study or not, (c) suggests that the research places participants at a greater risk of harm than previously known or not, and (d) is serious or not. Guidance for evaluating these is provided in our data and safety monitoring plan, which has been reviewed with each research staff member. When an AE report has been created in REDCap that suggests that an AE has occurred that is unexpected, related, and serious or suggests greater risk than previously known, the PI and study coordinator at the lead site are automatically emailed. The PI is responsible to ensure the reportable AEs are reported to the Data and Safety Monitoring Board, all IRBs, the study sponsor, and the Office of Human Research Protections in the timeframes established by federal guidelines. Given the low likelihood of serious risk in this study, the study has no provisions for compensating participants who suffer AEs, nor for any ancillary or post-trial care.

In addition to this first level of AE monitoring, the lead site reviews all AEs across all study sites at quarterly meetings of the MetroHealth Rehabilitation Institute AE Review Committee. This committee includes PIs and study staff involved in stroke rehabilitation clinical trials active at MetroHealth. At these meetings, all AEs are reviewed and consensus is reached as to their classification as related or not, unexpected or not, and serious or not.

### Study management and oversight

The study is being conducted by a multidisciplinary research team across four sites: (1) MetroHealth Center for Rehabilitation Research (MCRR) and the Cleveland Clinic (CC), (2) Kessler Foundation (KF), (3) Johns Hopkins University (JHU), and (4) Emory University and the Atlanta VA (EU-AVA). MCRR is the lead site. The principal investigator (PI) at the lead site is Jayme Knutson, PhD, who has overall responsibility for the execution and reporting of the trial. He is the primary contact PI for the study, assumes fiscal and administrative responsibility for the study, and submits annual progress reports to the funding agency. Each site has a site PI, A.M. Barrett, MD at EM-AVA, Preeti Raghavan, MD at JHU, Olga Boukrina at KF, and Ela B. Plow, PhD at CC. Each site PI is responsible for managing their subcontract and providing leadership at their site, including overseeing their study staff, and ensuring study activities are being conducted according to the study protocol and regulatory (i.e., IRB) requirements at their site, including annual continuing review submissions. In addition, John Chae, MD is the study physician at the lead site and is responsible for medical oversight of the study, including consulting on issues regarding AEs and evaluating eligibility criteria. Doug Gunzler, PhD is the study statistician and is responsible for data analysis and reporting to the study’s data and safety monitoring board (DSMB) annually.

Each site has study coordinator, who is responsible under the oversight of their site PI to manage the day-to-day operations of the study at their site. Each study coordinator is responsible for preparing IRB correspondence, recruiting participants and meeting enrollment targets, reporting screening and enrollment progress monthly, and attending monthly study management meetings hosted by the lead site. The study coordinator at the lead site has additional responsibilities under the direction of the lead PI that include developing training materials and training study coordinators at all sites; creating and updating the manual of procedures and REDCap data collection forms; purchasing supplies and shipping stimulators, therapy supplies, and assessment instruments to each site; responding to questions from site study coordinators; assisting the study statistician in creating DSMB reports; receiving monthly screening and enrollment data from study sites; and creating monthly study management meeting agendas. The treating and assessing therapists at the lead site are responsible for creating training materials, training their counterparts at the other study sites, ensuring certifications of treating and assessing therapists are up to date, and responding to questions from site therapists regarding administration of treatments and assessments.

The monthly study management meeting is held by videoconferencing and is attended by the site PIs and study coordinators. It is led by the lead site coordinator and the study PI. The agenda covers enrollment progress, any protocol amendments, re-credentialing reminders, any changes in data collection forms, issues related to treatment or assessments, and any other issues or questions that have emerged. Any protocol modifications are communicated to site PIs and study coordinators at this meeting.

A DSMB has been established for this study that is comprised of 5 members: two stroke physiatrists, an occupational therapist, a biomedical engineer, and a biostatistician. Their purpose is to monitor the safety of the participants and the scientific integrity of the study. The annual DSMB meeting includes a brief update of study activities, challenges, and progress since the last meeting presented by the lead site PI, presentation and discussion of a report prepared by the study statistician with the assistance of the study coordinator at the lead site, and closed discussion of the report by DSMB members only during which they decide on recommendations they will make to the PI regarding the continuation of the study with or without modifications. The DSMB report includes number of potential study participants screened and enrolled per site; number of participants who withdrew before end of treatment (and reasons) per site and per blinded group assignment; descriptions of any major protocol deviations; demographics of enrolled participants by blinded group assignment; number, description, and classification of adverse events sorted by study site and per blinded group assignment; and adherence to the treatment visits and home regimen by site and by blinded group assignment. The DSMB sends their recommendations to the PI, which may include their evaluation of the benefit/risk ratio of procedures and participant burden; selection, recruitment, and retention of participants; maintenance of equipoise; and safety concerns. The DSMB recommendations are reported to the IRBs at all sites and to the study sponsor (NIH).

### Dissemination plan

This protocol is registered in ClinicalTrials.gov (NCT03574623). The results of the trial will be reported no later than 1 year after the trial’s primary completion date. Study participants and the public will be able to access the study results through ClincalTrials.gov. In addition, the results of this research will be submitted for publication in international peer-reviewed journals and for oral and/or poster presentations at national and international conferences. Both positive and negative results will be reported. Privacy of participant-level clinical data will be maintained and not published.

## Discussion

The need is great for stroke rehabilitation treatments that are more effective in restoring upper limb function and that can be delivered at high doses without requiring very expensive equipment and extensive training or specialized personnel. Stroke rehabilitation technologies and treatments must progress through multiple phases of research and development before they reach clinical acceptance and are translated to clinical practice. Progressing from single-site to multi-site clinical trials is a critical step in the process. Multi-site studies prove whether a technology and/or treatment can be successfully administered by sites other than the originating site and with similar positive outcomes. This study is the first multi-site clinical trial of CCFES. As such, it is a critical step in advancing a novel method of rehabilitation toward clinical translation and widespread dissemination. More generally, this study will contribute to our knowledge of upper extremity recovery after stroke, how to incorporate neurotechnology into therapy, and how to successfully administer therapies that have significant (10 h/week × 12 weeks) self-administered home regimens.

This multi-site trial will have several advantages over prior CCFES studies. First, adding sites that are not familiar with CCFES will reduce biases that may be present at the lead site, which has been studying CCFES for more than a decade, and will determine whether positive results can be achieved regardless of site. Second, it will have a larger sample size of the target subpopulation, which will allow more precise determination of effect sizes. Third, it will generate valuable information from therapists regarding the ease of deploying the technology and recommendations for device revisions that would be necessary for widespread clinical acceptance.

Each of the three specific aims adds unique value to this study. The first aim, to confirm and demonstrate the generalizability of the efficacy of CCFES by comparing CCFES to cNMES will determine whether the CCFES method of delivering electrical stimulation really matters (explanatory aim). But perhaps of greater practical importance is the second aim, which will determine how CCFES compares to TOT, a treatment that has greater resemblance to usual care than cNMES (pragmatic aim). The third aim seeks to extend initial investigations of the neuroplastic mechanisms of CCFES (exploratory aim) [59]. Comparing the effects that CCFES, cNMES, and TOT have on interhemispheric and corticospinal activity will provide data that may help us understand the different effects of bilateral vs. unilateral therapy.

### Trial status

Recruitment started in February 2019. The study is currently enrolling participants. Enrollment is expected to close in 2023.

## Supplementary Information

**Additional file 1.**

## Data Availability

This manuscript does not include any data.

## Abbreviations

ADL: Activities of daily living
AE: Adverse event
AMAT: Arm Motor Ability Test
ARAT: Action Research Arm Test
BBT: Box and Blocks Test
CCFES: Contralaterally controlled functional electrical stimulation
cNMES: Cyclic neuromuscular electrical stimulation
DSMB: Data and Safety Monitoring Board
EDC: Extensor digitorum communis
EU-AVA: Emory University-Atlanta Veterans Affairs
FDA: Food and Drug Administration
GRASP: Graded Repetitive Arm Supplementary Program
HIPAA: Health Insurance Portability and Accountability Act
ICF: International Classification of Functioning and Disability Framework
IRB: Institutional Review Board
JHU: Johns Hopkins University
KF: Kessler Foundation
LTP: Long-term potentiation
MCID: Minimal clinically important difference
MCRR: MetroHealth Center for Rehabilitation Research
MDC: Minimum detectable change
MEP: Motor evoked potential
MOP: Manual of procedures
NMES: Neuromuscular electrical stimulation
QOL: Quality of life
RCT: Randomized controlled trial
SIS: Stroke Impact Scale
SULCS: Stroke Upper Limb Capacity Scale
TMS: Transcranial magnetic stimulation
TOT: Task-oriented training
UEFM: Upper Extremity Fugl-Meyer

## References

[1] Virani SS, Alonso A, Aparicio HJ, Benjamin EJ, Bittencourt MS, Callaway CW (2021). Heart Disease and Stroke Statistics-2021 Update: a report from the American Heart Association. Circulation..

[2] Lai SM, Studenski S, Duncan PW, Perera S (2002). Persisting consequences of stroke measured by the Stroke Impact Scale. Stroke..

[3] Lang CE, DeJong SL, Beebe JA (2009). Recovery of thumb and finger extension and its relation to grasp performance after stroke. J Neurophysiol.

[4] Kamper DG, Fischer HC, Cruz EG, Rymer WZ (2006). Weakness is the primary contributor to finger impairment in chronic stroke. Arch Phys Med Rehabil.

[5] Franceschini M, La Porta F, Agosti M, Massucci M (2010). group ICR. Is health-related-quality of life of stroke patients influenced by neurological impairments at one year after stroke?. Eur J Phys Rehabil Med.

[6] Nudo RJ, Wise BM, SiFuentes F, Milliken GW (1996). Neural substrates for the effects of rehabilitative training on motor recovery after ischemic infarct. Science..

[7] Johansson BB (2000). Brain plasticity and stroke rehabilitation. Willis Lecture Stroke.

[8] Jenkins WM, Merzenich MM (1987). Reorganization of neocortical representations after brain injury: a neurophysiological model of the bases of recovery from stroke. Prog Brain Res.

[9] Merzenich MM, Jenkins WM (1993). Reorganization of cortical representations of the hand following alterations of skin inputs induced by nerve injury, skin island transfers, and experience. J Hand Ther.

[10] Merzenich MM, Kaas JH, Wall J, Nelson RJ, Sur M, Felleman D (1983). Topographic reorganization of somatosensory cortical areas 3b and 1 in adult monkeys following restricted deafferentation. Neuroscience..

[11] Merzenich MM, Nelson RJ, Stryker MP, Cynader MS, Schoppmann A, Zook JM (1984). Somatosensory cortical map changes following digit amputation in adult monkeys. J Comp Neurol.

[12] Kew JJ, Ridding MC, Rothwell JC, Passingham RE, Leigh PN, Sooriakumaran S (1994). Reorganization of cortical blood flow and transcranial magnetic stimulation maps in human subjects after upper limb amputation. J Neurophysiol.

[13] Mogilner A, Grossman JA, Ribary U, Joliot M, Volkmann J, Rapaport D (1993). Somatosensory cortical plasticity in adult humans revealed by magnetoencephalography. Proc Natl Acad Sci U S A.

[14] Pascual-Leone A, Torres F (1993). Plasticity of the sensorimotor cortex representation of the reading finger in Braille readers. Brain..

[15] Buonomano DV, Merzenich MM (1998). Cortical plasticity: from synapses to maps. Annu Rev Neurosci.

[16] Malenka RC, Nicoll RA (1999). Long-term potentiation--a decade of progress?. Science..

[17] Knutson JS, Harley MY, Hisel TZ, Chae J (2007). Improving hand function in stroke survivors: a pilot study of contralaterally controlled functional electric stimulation in chronic hemiplegia. Arch Phys Med Rehabil.

[18] Knutson JS, Gunzler DD, Wilson RD, Chae J (2016). Contralaterally controlled functional electrical stimulation improves hand dexterity in chronic hemiparesis: a randomized trial. Stroke..

[19] Knutson JS, Harley MY, Hisel TZ, Hogan SD, Maloney MM, Chae J (2012). Contralaterally controlled functional electrical stimulation for upper extremity hemiplegia: an early-phase randomized clinical trial in subacute stroke patients. Neurorehabil Neural Repair.

[20] Knutson JS, Hisel TZ, Harley MY, Chae J (2009). A novel functional electrical stimulation treatment for recovery of hand function in hemiplegia: 12-week pilot study. Neurorehabil Neural Repair.

[21] Knutson JS, Makowski NS, Harley MY, Hisel TZ, Gunzler DD, Wilson RD (2020). Adding contralaterally controlled electrical stimulation of the triceps to contralaterally controlled functional electrical stimulation of the finger extensors reduces upper limb impairment and improves reachable workspace but not dexterity: a randomized controlled trial. Am J Phys Med Rehabil.

[22] Zheng Y, Mao M, Cao Y, Lu X (2019). Contralaterally controlled functional electrical stimulation improves wrist dorsiflexion and upper limb function in patients with early-phase stroke: A randomized controlled trial. J Rehabil Med.

[23] Shen Y, Yin Z, Fan Y, Chen CF, Dai W, Yi W (2015). Comparison of the effects of contralaterally controlled functional electrical stimulation and neuromuscular electrical stimulation on upper extremity functions in patients with stroke. CNS Neurol Disord Drug Targets.

[24] Nascimento LR, Michaelsen SM, Ada L, Polese JC, Teixeira-Salmela LF (2014). Cyclical electrical stimulation increases strength and improves activity after stroke: a systematic review. J Physiother.

[25] Chae J, Bethoux F, Bohine T, Dobos L, Davis T, Friedl A (1998). Neuromuscular stimulation for upper extremity motor and functional recovery in acute hemiplegia. Stroke..

[26] Hsu SS, Hu MH, Wang YH, Yip PK, Chiu JW, Hsieh CL (2010). Dose-response relation between neuromuscular electrical stimulation and upper-extremity function in patients with stroke. Stroke..

[27] Knutson JS, Fu MJ, Sheffler LR, Chae J (2015). Neuromuscular electrical stimulation for motor restoration in hemiplegia. Phys Med Rehabil Clin N Am.

[28] Harris JE, Eng JJ, Miller WC, Dawson AS (2009). A self-administered Graded Repetitive Arm Supplementary Program (GRASP) improves arm function during inpatient stroke rehabilitation: a multi-site randomized controlled trial. Stroke..

[29] Organization WH (2013). How to use the ICF: a practical manual for using the International Classification of Funcitoning, Disability and Health (ICF).

[30] Database RM (2016). Rehabilitation Measures Database. Rehab Measures: Box and Block Test.

[31] Salter K, Campbell N, Richardson M, Mehta S, Jutai J, Zettler L, et al. Chapter 21. Outcome Measures in Stroke Rehabilitation. In: Teasell R, editor. Evidence-Based Review of Stroke Rehabilitation 16th Edition. London; 2013. www.ebrsr.com.

[32] Mathiowetz V, Volland G, Kashman N, Weber K (1985). Adult norms for the Box and Block Test of manual dexterity. Am J Occup Ther.

[33] Desrosiers J, Bravo G, Hebert R, Dutil E, Mercier L (1994). Validation of the Box and Block Test as a measure of dexterity of elderly people: reliability, validity, and norms studies. Arch Phys Med Rehabil.

[34] Chen HM, Chen CC, Hsueh IP, Huang SL, Hsieh CL (2009). Test-retest reproducibility and smallest real difference of 5 hand function tests in patients with stroke. Neurorehabil Neural Repair.

[35] Ghaziani E, Couppe C, Henkel C, Siersma V, Sondergaard M, Christensen H (2017). Electrical somatosensory stimulation followed by motor training of the paretic upper limb in acute stroke: study protocol for a randomized controlled trial. Trials..

[36] Page SJ, Fulk GD, Boyne P (2012). Clinically important differences for the upper-extremity Fugl-Meyer Scale in people with minimal to moderate impairment due to chronic stroke. Phys Ther.

[37] Fugl-Meyer AR, Jaasko L, Leyman I, Olsson S, Steglind S (1975). The post-stroke hemiplegic patient. 1. a method for evaluation of physical performance. Scand J Rehabil Med.

[38] Duncan PW, Propst M, Nelson SG (1983). Reliability of the Fugl-Meyer assessment of sensorimotor recovery following cerebrovascular accident. Phys Ther.

[39] Roorda LD, Houwink A, Smits W, Molenaar IW, Geurts AC (2011). Measuring upper limb capacity in poststroke patients: development, fit of the monotone homogeneity model, unidimensionality, fit of the double monotonicity model, differential item functioning, internal consistency, and feasibility of the stroke upper limb capacity scale. SULCS Arch Phys Med Rehabil.

[40] Houwink A, Roorda LD, Smits W, Molenaar IW, Geurts AC (2011). Measuring upper limb capacity in patients after stroke: reliability and validity of the stroke upper limb capacity scale. Arch Phys Med Rehabil.

[41] Knutson JS, Friedl AS, Hansen KM, Hisel TZ, Harley MY (2019). Convergent validity and responsiveness of the SULCS. Arch Phys Med Rehabil.

[42] Platz T, Pinkowski C, van Wijck F, Kim IH, di Bella P, Johnson G (2005). Reliability and validity of arm function assessment with standardized guidelines for the Fugl-Meyer Test, Action Research Arm Test and Box and Block Test: a multicentre study. Clin Rehabil.

[43] Yozbatiran N, Der-Yeghiaian L, Cramer SC (2008). A standardized approach to performing the action research arm test. Neurorehabil Neural Repair.

[44] Hsieh CL, Hsueh IP, Chiang FM, Lin PH (1998). Inter-rater reliability and validity of the action research arm test in stroke patients. Age Ageing.

[45] Bohannon RW, Smith MB (1987). Interrater reliability of a modified Ashworth scale of muscle spasticity. Phys Ther.

[46] Duncan PW, Bode RK, Min Lai S, Perera S (2003). Glycine antagonist in neuroprotection Americans I. Rasch analysis of a new stroke-specific outcome scale: the Stroke Impact Scale. Arch Phys Med Rehabil.

[47] Cella D, Lai JS, Nowinski CJ, Victorson D, Peterman A, Miller D (2012). Neuro-QOL: brief measures of health-related quality of life for clinical research in neurology. Neurology..

[48] Cunningham DA, Machado A, Janini D, Varnerin N, Bonnett C, Yue G (2015). Assessment of inter-hemispheric imbalance using imaging and noninvasive brain stimulation in patients with chronic stroke. Arch Phys Med Rehabil.

[49] Tazoe T, Perez MA (2014). Selective activation of ipsilateral motor pathways in intact humans. J Neurosci.

[50] Perez MA, Cohen LG (2009). Scaling of motor cortical excitability during unimanual force generation. Cortex.

[51] Taves DR (1974). Minimization: a new method of assigning patients to treatment and control groups. Clin Pharmacol Ther.

[52] Pocock SJ, Simon R (1975). Sequential treatment assignment with balancing for prognostic factors in the controlled clinical trial. Biometrics..

[53] Wolf SL, Winstein CJ, Miller JP, Taub E, Uswatte G, Morris D (2006). Effect of constraint-induced movement therapy on upper extremity function 3 to 9 months after stroke: the EXCITE randomized clinical trial. Jama..

[54] Connell LA, McMahon NE, Harris JE, Watkins CL, Eng JJ (2014). A formative evaluation of the implementation of an upper limb stroke rehabilitation intervention in clinical practice: a qualitative interview study. Implement Sci.

[55] Harris PA, Taylor R, Minor BL, Elliott V, Fernandez M, O'Neal L (2019). The REDCap consortium: Building an international community of software platform partners. J Biomed Inform.

[56] Harris PA, Taylor R, Thielke R, Payne J, Gonzalez N, Conde JG (2009). Research electronic data capture (REDCap)--a metadata-driven methodology and workflow process for providing translational research informatics support. J Biomed Inform.

[57] Fitzmaurice GM, Laird NM, Ware JH (2012). Applied longitudinal analysis.

[58] Baron RM, Kenny DA (1986). The moderator-mediator variable distinction in social psychological research: conceptual, strategic, and statistical considerations. J Pers Soc Psychol.

[59] Cunningham DA, Knutson JS, Sankarasubramanian V, Potter-Baker KA, Machado AG, Plow EB (2019). Bilateral contralaterally controlled functional electrical stimulation reveals new insights into the interhemispheric competition model in chronic stroke. Neurorehabil Neural Repair.

